# Symptom-based diagnostic approach for eosinophilic esophagitis

**DOI:** 10.1007/s00535-020-01701-y

**Published:** 2020-07-27

**Authors:** Yasuhiro Fujiwara

**Affiliations:** Department of Gastroenterology, Osaka City University Graduate School of Medicine, 1-4-3 Asahimachi, Abenoku, Osaka 545-8585 Japan

**Keywords:** Eosinophilic esophagitis, Esophageal eosinophilia, Dysphagia

## Abstract

The prevalence of eosinophilic esophagitis (EoE) has been increasing in Japan. Although the diagnosis of EoE is based on simple criteria that include the presence of esophageal symptoms and esophageal eosinophilia on biopsies, there are several important issues associated with the diagnostic approach. Following an extensive literature search, the symptoms of 886 EoE cases in Japanese adults were analyzed and divided into three categories as follows: (1) typical symptoms, such as dysphagia (53%); (2) other upper GI symptoms (40%); and (3) no symptoms found during screening or medical examination, i.e., “asymptomatic esophageal eosinophilia” (19%). The diagnostic approach was reviewed according to these categories as well as according to the presence or absence of esophageal eosinophilia. The present manuscript describes the current therapeutic strategy of EoE and ultimately proposes a symptom-based diagnostic approach for EoE.

## Introduction

Eosinophilic esophagitis (EoE) is a chronic antigen-mediated allergic disease of the esophagus in adults and children [1–3]. EoE is commonly found in Western countries but is relatively rare in Asia [4, 5]; nevertheless, the prevalence of EoE has been increasing in Japan [6–8]. Recent European guidelines [2] and International consensus (AGREE) [3] have demonstrated that the diagnostic criteria of EoE include the presence of esophageal symptoms and esophageal eosinophil (eos) infiltration, defined as intraepithelial eosinophil infiltration of ≥ 15 eos/high-power field (hpf). Although the diagnosis of EoE is not clinically challenging, several important issues require consideration in the diagnostic approach. Most patients in Western countries complain of typical symptoms such as dysphagia and food impaction [9, 10]; however, the prevalence of symptom variations in Japanese adult patients with EoE has not yet been elucidated. The current study analyzed the prevalence of symptoms in Japanese patients with EoE, categorized the symptoms, provided a review of the diagnostic approach, briefly explained the current therapeutic strategies, and finally proposed a symptom-based diagnostic approach. Although the current criteria require the presence of esophageal symptoms [1–3], asymptomatic cases have been reported in Japan, particularly during medical examinations [11–14]. Therefore, asymptomatic cases, formally known as “asymptomatic esophageal eosinophilia,” have been included in this review as asymptomatic EoE.

## Prevalence of symptom variations

Clinical studies and case reports of EoE in Japanese adults were identified by searching PubMed, Ichushi, Shoreikun, and UMIN in February 2020. Relevant articles were identified using terms “Japanese” or “Japan” and “eosinophilic esophagitis” or “esophageal eosinophilia”. Articles and abstracts of pediatric cases as well as basic studies using animals or cell lines were excluded. Clinical studies or case reports and abstracts of case reports or case series that included clinical symptoms and their prevalence were selected. Overall, 53 full articles, including 31 case reports [11–63] and 60 abstracts (in Japanese), were collected and reviewed. The symptoms were divided into three categories as follows: (1) typical symptoms, such as dysphagia and food impaction; (2) other upper GI symptoms, such as heartburn, acid regurgitation, chest pain, epigastralgia and abdominal pain, nausea or vomiting, globus sensation, odynophagia, and anorexia; and (3) no symptoms found incidentally during screening or medical examinations.

**Fig. 1:**
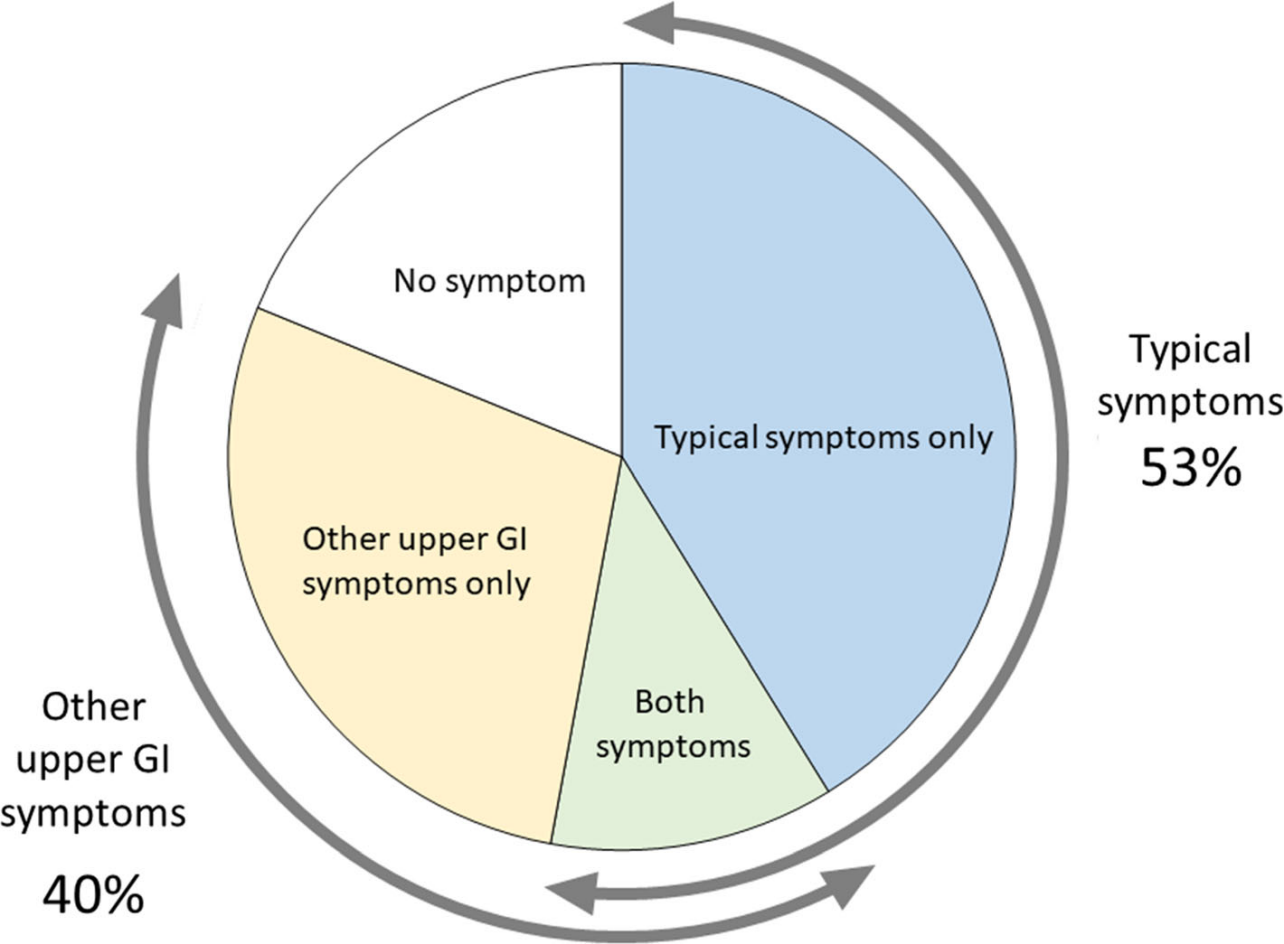
Proportion of symptoms in Japanese adults with eosinophilic esophagitis. Symptoms were divided into typical symptoms (such as dysphagia and food impaction), other upper GI symptoms (such as heartburn, epigastralgia, and chest pain), and no symptoms

The analysis of 886 cases is shown in Table 1. Typical symptoms, such as dysphagia or food impaction, were observed in 469 (52.9%) cases, whereas heartburn or acid regurgitation was noted in 224 (25.3%), chest pain in 59 (6.7%), epigastralgia or abdominal pain in 42 (4.7%), other symptoms in 38 (4.3%), and no symptoms in 167 (18.8%) cases. Figure 1 shows the proportion of symptoms according to the symptom categories of EoE in Japanese patients. Typical symptoms were found in 41.2% of cases, other upper GI symptoms were found in 28.3%, both symptoms were found in 11.7%, and no symptoms were found in 18.8%. Western studies have shown that dysphagia (70–80%) and food impaction (33–54%) constitute the most common symptoms of adult EoE [2, 9, 10]. The present study showed similar results, but the prevalence of typical symptoms was lower than that observed in Western countries. Dellon et al. analyzed the symptoms of 151 adults with EoE and reported the presence of dysphagia (73%), food impaction (30%), heartburn (42%), chest pain (8%), and abdominal pain (26%) [9], suggesting that the prevalence of heartburn and abdominal pain in Japanese patients with EoE was low: when EoE patients with symptoms were analyzed, prevalence of dysphagia/food impaction, heartburn/acid regurgitation, chest pain, and epigastralgia/abdominal pain was 65.2%, 31.2%, 8.2%, and 5.8%, respectively.

**Table 1:** Prevalence of symptoms of eosinophilic esophagitis in Japanese adults

Author [Ref]	Number of cases	Dysphagia food impaction	Heartburn/acid regurgitation	Chest pain/chest discomfort	Epigastralgia/Abdominal pain	Others symptoms*	Other upper GI symptoms**	No symptoms
Abe et al. [15]	12	7	1	1	2	0	4	3
Fujishiro et al. [16]	7	2	2	0	2	0	4	0
Fujiwara et al. [17]	7	3	3	0	0	0	3	1
Kinoshita et al.[18]	26	12	2	0	0	13	15	0
Tomomatsu et al.[19]	10	6	3	1	0	0	4	0
Hori et al. [20]	5	2	0	0	0	1	1	2
Abe et al. [15]	10	1	2	0	0	0	2	8
Shimura et al. [22]	12	5	4	1	1	0	6	4
Kusunose et al.[23]	13	8	6	0	1	0	7	0
Iwakura et al. [24]	33	30	6	4	0	0	10	0
Ishimura et al. [25]	4	3	2	0	0	0	2	0
Jiao et al. [26]	27	15	13	4	0	5	22	0
Okimoto et al. [27]	70	37	22	0	13	0	35	9
Okimoto et al. [28]	6	2	3	0	1	0	4	0
Adachi et al. [11]	36	17	12	1	1	0	14	11
Sato et al. [12]	17	5	5	0	0	0	5	7
Ishimura et al. [29]	55	31	18	0	0	0	18	0
Ishimura et al. [30]	147	76	52	16	0	0	68	25
Sawada et al. [31]	106	73	27	16	0	0	43	12
Takashima et al. [32]	27	19	1	0	0	0	1	7
Tanaka et al. [13]	27	19	1	0	0	0	1	7
Ishibashi et al. [14]	37	0	0	0	0	0	0	37
Case reports#	39	20	3	5	5	6	17	8
Abstracts##	153	76	36	10	16	13	68	26
Total	886	469	224	59	42	38	354	167
Prevalence	100%	52.9%	25.3%	6.7%	4.7%	4.3%	40.0%	18.8%
Patients with symptoms	719	469	224	59	42	38	354	
Prevalence###	100%	65.2%	31.2%	8.2%	5.8%	5.3%	49.2%	

*Other symptoms include nausea/vomiting, globus sensation, anorexia, odynophagia, and belching

** Number of other upper GI symptoms is the incidence of heartburn/acid regurgitation, chest pain/discomfort, epigastralgia/abdominal pain, and other symptoms

# Summary of 31 full articles of case reports

## Summary of 70 abstracts of case reports and series

### prevalence calculated in patients with symptoms

Further, seven case series and 91 case reports and abstracts describing patient age and sex were collected. Finally, 131 patient cases (91 men and 40 women; mean age, 49.8 years) were analyzed in detail according to age and sex. In this review, patient ages were divided into 3 groups as follows: young (20–39 years), middle (40–69), and elderly (≥ 70 years). Among the 131 cases, 34 were young, 80 were middle-aged, and 17 were elderly. Figure 2 shows the prevalence of various symptoms according to the age group. Dysphagia or food impaction was the most common symptom across the age groups. There was a significant increase in the incidence of chest pain or discomfort in the middle-aged group and in the incidence of anorexia in the elderly, as determined using Chi-square test. However, there were no significant differences in the other symptoms, such as reflux symptoms and epigastralgia, among the age groups. Some differences in symptom patterns among age groups were observed between Western and Japanese patients. Indeed, Dellon et al. showed that other upper GI symptoms were observed in younger patients, whereas typical symptoms were primarily observed in comparatively older patients [9]. There were no sex differences in relation to the prevalence of symptoms (dysphagia 65.9% in men and 62.5% in women, heartburn/regurgitation 25.3% in men and 27.5% in women, epigastralgia/abdominal pain 8.8% in men and 17.5% in women, chest pain/discomfort 14.3% in men and 20.0% in women, nausea/vomiting 3.3% in men and 5.0% in women, globus 3.3% in men and 0% in women, anorexia 2.2% in men and 2.5% in women, and odynophagia 0% in men and 2.5% in women). These results suggest that in the diagnosis of EoE, physicians should pay close attention to chest pain in middle-aged patients and anorexia in elderly patients. Moreover, we demonstrated that dysphagia/food impaction, heartburn/acid regurgitation, and epigastralgia/abdominal pain are common symptoms in Japanese patients with EoE although food impaction requiring emergency endoscopy is uncommon in Japan.

**Fig. 2:**
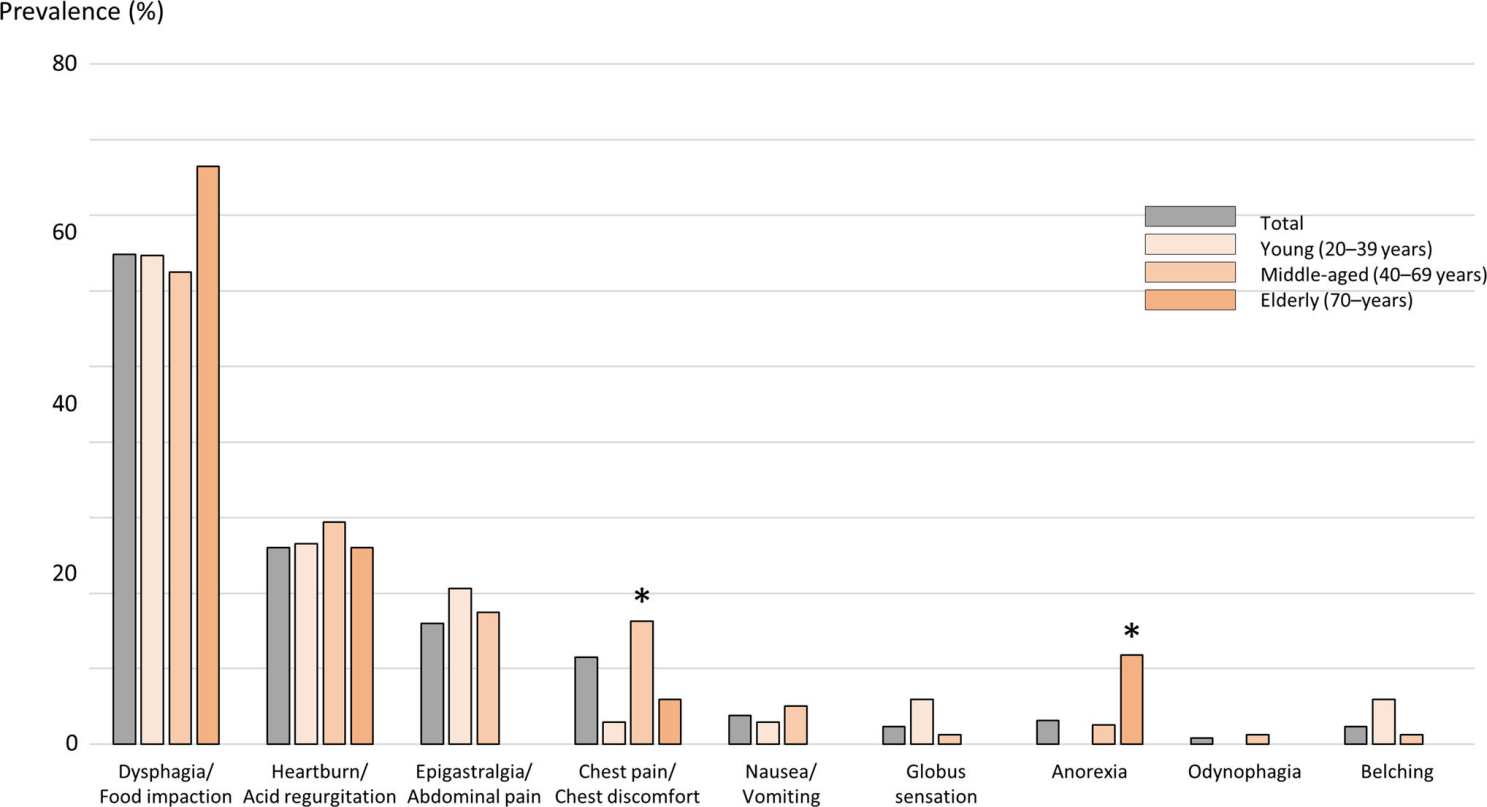
Prevalence of various symptoms in Japanese adults with eosinophilic esophagitis according to age group, Chest pain or discomfort was significantly more common in the middle-aged group, and anorexia was significantly more common in the elderly group. The prevalence of other symptoms was similar among the age groups (*p < 0.01)

## Diagnostic approach-1

The diagnostic approach was described according to symptom categories.

### Typical symptoms

Dysphagia and food impaction are caused by several diseases; in particular, esophageal cancers, gastroesophageal reflux disease (GERD), and achalasia are important and should be excluded. Several guidelines have proposed that biopsies should be obtained in cases where EoE is suspected from symptoms regardless of endoscopic findings [1–3]. European guidelines state that at least six biopsies should be performed from different locations, focusing on areas with endoscopic mucosal abnormalities [2]. Moreover, the American College of Gastroenterology guidelines strongly recommends that 2–4 biopsies should be obtained from both the proximal and distal esophagus [1]. In our previous study [64], two biopsies each from the lower and middle esophagus revealed a high diagnostic accuracy rate. Therefore, multiple biopsies (4–6) should be recommended in patients with typical symptoms.

**Fig. 3:**
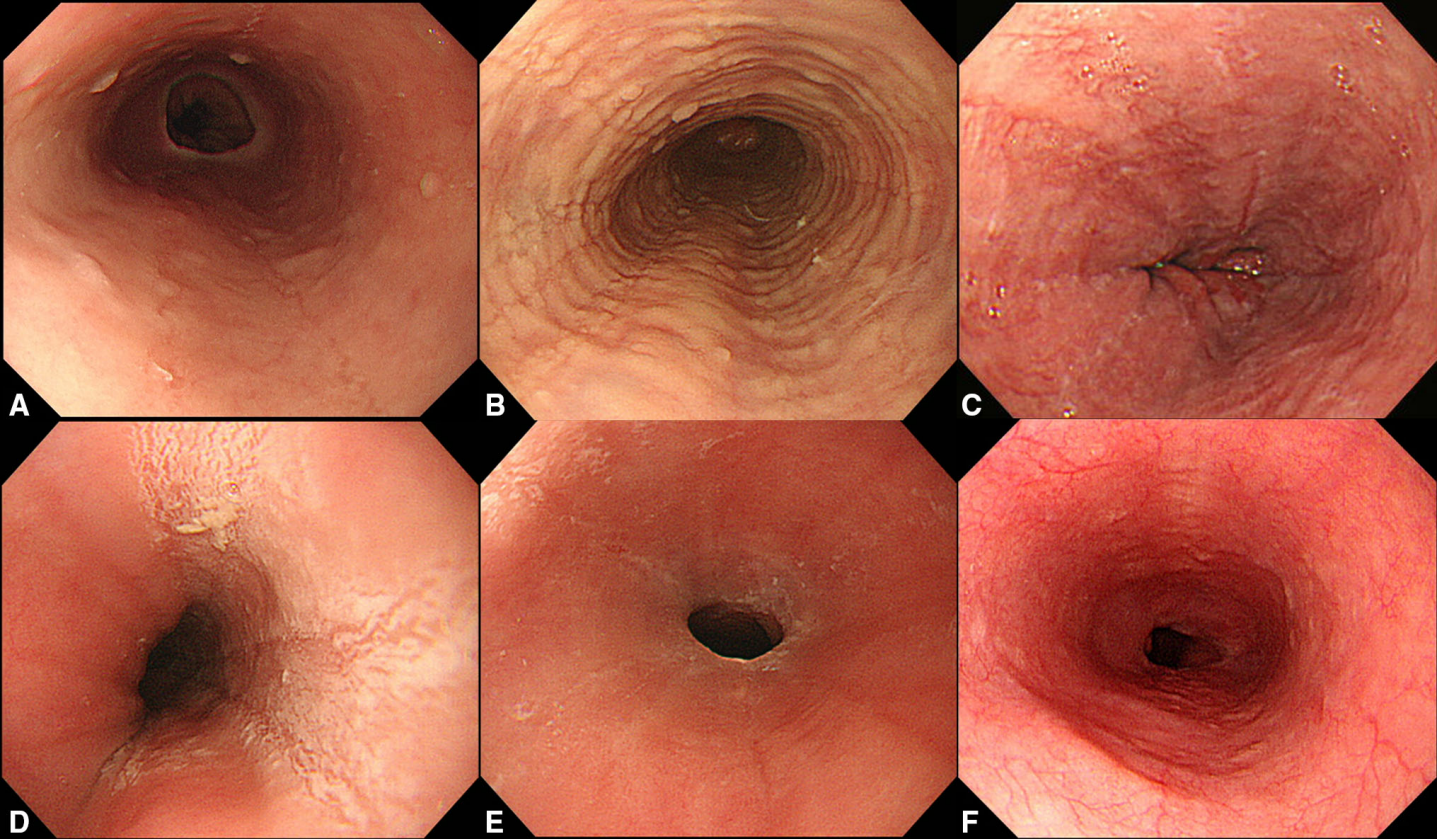
Endoscopic appearance of eosinophilic esophagitis. **a** Edema, grade 1. **b** Rings, grade 1. **c** Furrows, grade 1. **d** Exudates, grade 2. **e** Stricture, grade 1. **f** Normal appearance. Endoscopic grading was performed according to the EREFS score system

Typical endoscopic findings include edema, rings, exudates, furrows, and strictures [1–3] (Fig. 3). The endoscopic findings were graded by the Endoscopic Reference Score System (ERERS) as follows: edema (0, absent; 1, present); rings (0, none; 1, mild; 2, moderate; 3, severe); exudates [0, none; 1, mild (< 10% of the esophageal surface area); 2, severe (> 10% of the esophageal surface area)]; furrows (0, absent; 1, present); and strictures (0, absent; 1, present) [65]. Although fibrostenotic cases with rings or strictures are clinically problematic and common in Western countries [66], grade 3 rings and grade 1 strictures are currently rare in Japanese patients. Although Japanese endoscopists prefer endoscopic diagnosis, there are several important issues with regard to such diagnosis of EoE. First, Izumi et al. reported that the inter-observer agreement on the endoscopic diagnosis of EoE among Japanese endoscopists was low and revealed that the kappa coefficient of reliability was 0.34 (0.33–0.35) [67]. Second, three previous studies have shown that the prevalence of esophageal eosinophilia on biopsy in cases with typical endoscopic EoE findings was low (11.1–30.8%) [20, 22, 68]. This is related to the international diagnostic criteria that do not include endoscopic findings. Third, although several guidelines have proposed multiple biopsies of normal esophageal mucosal appearance in cases suspicious of EoE [1, 3], a Japanese multicenter study only found 1 such case (0.34%) in a total of 289 cases [22].

The aforementioned reports emphasize the importance of multiple biopsies in patients with typical symptoms. Indeed, multiple biopsies are the only modality that can exclude EoE when no significant esophageal eosinophilia is observed. Rome IV criteria suggest that exclusion of EoE is necessary for the diagnosis of functional dysphagia [69].

### Other upper GI symptoms

A similar diagnostic strategy to that illustrated above is adapted in patients with other upper GI symptoms, such as heartburn, acid regurgitation, epigastralgia, abdominal pain, nausea, vomiting, chest pain, and globus sensation. However, in cases with endoscopically normal esophageal appearance, multiple biopsies should be limited when patients are refractory to the standard treatment. EoE is one of the causes of proton pump inhibitor (PPI)-refractory GERD [70]. Kawami et al. analyzed 53 cases with double doses of PPI-resistant nonerosive reflux disease, defined as the presence of reflux symptoms without esophageal mucosal breaks, and found only 1 (1.9%) case of EoE [71]. Furthermore, Okimoto et al. performed multiple biopsies of the esophagus in 62 patients with PPI-refractory GERD. They found 6 (9.7%) cases with EoE, five of which had an endoscopically normal esophageal appearance [28].

The exact prevalence of EoE among PPI-refractory GERD in Japanese patients is unknown; thus, a large multicenter study is required. Finally, it is necessary to obtain multiple biopsies because the exclusion of EoE is one of the diagnostic criteria of functional heartburn, reflux hypersensitivity, functional chest pain, and globus according to the Rome IV criteria [69].

### Medical health examinations

The local government or employer provides annual medical examinations for inhabitants and employers in Japan. Because cancer screening programs, particularly for gastric cancer, are widely accepted in Japan, EoE is often incidentally found during esophagogastroduodenoscopy (EGD). Several studies have shown that the prevalence of EoE among individuals who underwent annual medical examination ranged from 0.06 to 0.47% [11–14]. However, whether biopsies should be obtained in cases with EoE endoscopic findings observed in medical examinations remains debatable because the positive rate of esophageal eosinophilia in cases with EoE endoscopic findings is low [20, 22, 68] and because most individuals who undergo medical examinations are healthy or have mild or no symptoms. Minimal target biopsies can be recommended in such cases for the following two reasons. First, it is difficult to perform a detailed medical interview for EoE before and during endoscopy. Although Ishibashi et al. reported that all 37 cases of EoE found in medical examinations were asymptomatic [14], 3 other studies showed that 64–74% of the EoE cases found in medical examinations had symptoms [11–13]. Although some symptoms were mild, others required treatment. Second, studies conducted in Western countries have suggested that untreated EoE is associated with persistent symptoms and inflammation, leading to esophageal remodeling and a fibrostenotic phenotype [2].

The natural history of Japanese EoE, especially asymptomatic EoE, has not yet been completely elucidated. Ishibashi et al. demonstrated that 18 (62.1%) of 29 cases with asymptomatic EoE developed progressive diseases, including six cases with symptomatic EoE and 12 cases with endoscopic exacerbation [14]. Sato et al. showed that most cases with asymptomatic EoE showed a slow progression as observed on chronological endoscopic analysis [12]. Therefore, biopsies might be necessary to examine the natural history of Japanese patients with asymptomatic EoE. Adachi et al. showed that significant esophageal eosinophilia could be detected by biopsies on the lower esophagus as well as by the presence of exudates [72]. In summary, target biopsies are recommended in cases that are endoscopically suspicious of EoE during medical examinations, particularly in cases with any mild symptoms or severe endoscopic findings that might be associated with a fibrostenotic phenotype. Furthermore, in asymptomatic EoE cases, it is not always necessary to perform a biopsy. Another option is to refer to a high-volume center for re-examination of endoscopy with multiple biopsies.

## Diagnostic approach-2

Diagnosis after biopsy examination according to the presence or absence of esophageal eosinophilia has been discussed.

### Esophageal eosinophilia

All cases with esophageal eosinophilia, defined as ≥ 15 eos/hpf (~ 60 eos/mm^2^), involve differential diagnoses, such as GERD, eosinophilic gastroenteritis, achalasia, viral or fungal infection, autoimmune diseases, skin diseases, drug-induced esophagitis, Crohn disease, and graft versus host disease [1–3]. Most cases can be differentiated from EoE via physical examination, blood tests, endoscopic appearance, and other diagnostic modalities. Nevertheless, the association between GERD and EoE remains controversial because PPI therapy has improved both diseases [2, 3] and because GERD is a common disease affecting 10–20% of Japanese adults [73] and may be overlapped with EoE. However, based on typical EoE endoscopic findings with esophageal eosinophilia on biopsies, the diagnosis of EoE is relatively straightforward.

**Fig. 4:**
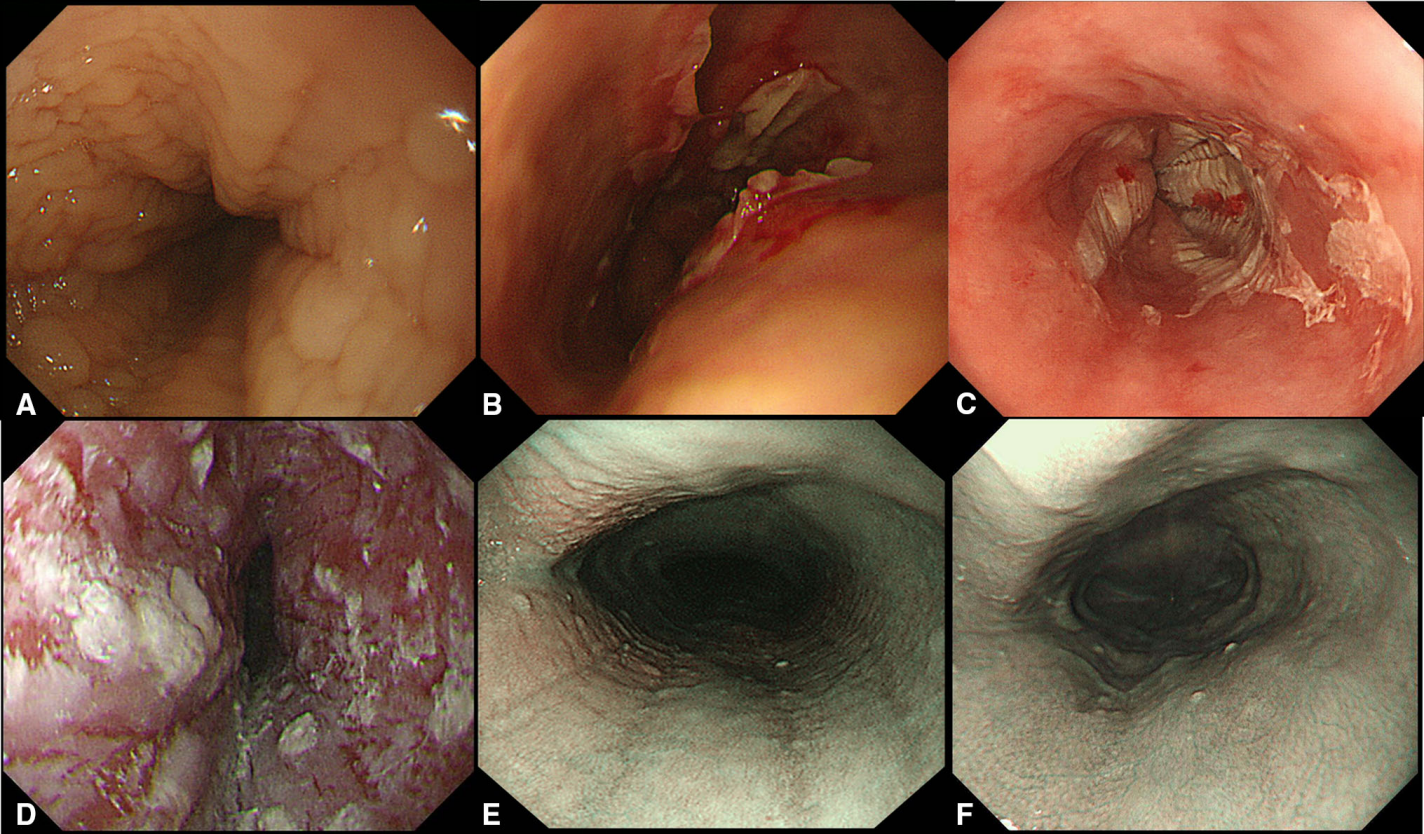
Cases with similar endoscopic appearance of eosinophilic esophagitis and special type of eosinophilic esophagitis. (**a**, **b**) Autoimmune esophagitis. **c** Pemphigoid esophagitis without skin lesions. **d** Dabigatran-induced esophagitis. **e**, **f** EoE caused by sublingual immunotherapy (SLIT). EoE findings (**e**) disappeared after avoiding swallowing medication during SLIT (**f**). NBI images are shown (**e**, **f**)

In differential diagnosis, physicians should pay attention to that some cases of esophageal eosinophilia have similar endoscopic findings of EoE (Fig. 4). Figure 4a–c reveals autoimmune esophagitis [74] and pemphigoid esophagitis without skin manifestations. In such cases, anti-Desmoglein (DSG)-1, anti-DSG-3, or anti-BP180 antibodies may assist in diagnosis. Nakamura et al. analyzed esophageal involvement in 123 autoimmune bullous diseases, and esophageal lesions, such as erosions, blisters, ulcers, and stenoses, were detected in 33 (26.8%) cases. They reported that approximately half of the cases had oral or laryngopharyngeal lesions and that the Nikolsky sign (epidermolysis by mechanical stimulus) is specific for autoimmune bullous diseases [75]. Figure 4d shows dabigatran-induced esophagitis, presenting as white exudates on the entire esophageal surface. Moreover, drug history can correctly diagnose EoE, suggesting the importance of medical interviews. Toyo et al. demonstrated that 19 (20.9%) of the 91 patients receiving dabigatran showed esophagitis and that longitudinally sloughing epithelial casts in the middle or lower esophagus was a typical endoscopic finding [76]. A special type of EoE caused by sublingual immunotherapy (SLIT) for cedar pollinosis is shown in Fig. 4e, f. Kawashima et al. reported a similar case successfully treated with PPI administration [56]. In this case, avoiding swallowing medication during SLIT for 2 months improved the patient’s symptoms, endoscopic appearance, and histological esophageal eosinophilia. Medications (such as dabigatran and SLIT) and blood tests (such as those with anti-DSG-1, DSG-3, and BP180 antibodies) may help in the differential diagnosis of EoE in some cases.

### Absence of esophageal eosinophilia

**Fig. 5:**
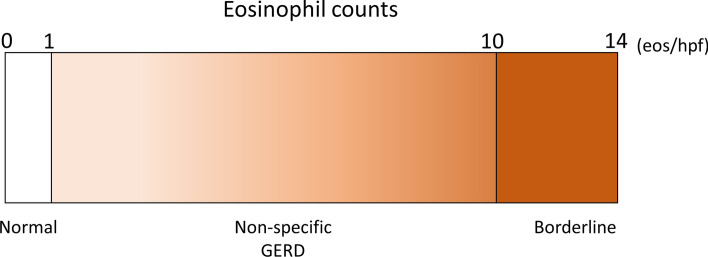
Concept of eosinophil infiltration in cases without esophageal eosinophilia. Esophageal eosinophilia was defined as ≥ 15 eos/hpf, whereas normal esophagus ranged from 0 to 1 eos/hpf. Eosinophil counts between 2 and 9 eos/hpf were considered as GERD or non-specific esophagitis and those between 10 and 14 eos/hpf were considered borderline esophageal eosinophilia

Matsushita et al. analyzed the number of eosinophils in the GI tract of healthy adults and found that normal esophageal eosinophil infiltration was 0.07 ± 0.43 (mean ± SD)/mm^2^, revealing that the normal limit of esophageal eosinophils is 0–1 eos/hpf [77]. It remains unknown whether cases with the number of esophageal eosinophils between 2 and 14 eos/hpf represent the same condition. It can be considered that cases with ≥ 10 eos/hpf were borderline EoE and require re-evaluation of biopsy specimens and careful follow-up, because patchy eosinophils infiltrate into the esophageal epithelium. Other cases with 2–9 eos/hpf were considered non-allergic, and such cases may be diagnosed as GERD or non-specific esophagitis. Figure 5 shows the concept of the number of esophageal eosinophils being < 15 eos/hpf. It is necessary to receive re-examination of biopsy or carful follow-up when EoE is highly suspected in case with the absence of esophageal eosinophilia.

The diagnosis of cases with the absence of esophageal eosinophilia has been described as a point-by-point discussion.

#### On-PPI or off-PPI

Researchers have carefully assessed cases without esophageal eosinophilia with normal biopsy results and endoscopic findings on PPI therapy [3]. Odiase et al. reported 2 EoE cases in whom PPI treatment totally obliterated the endoscopic and histologic evidence of EoE. They emphasized that PPI therapy should be discontinued 3–4 weeks before EGD to minimize diagnostic errors of EoE [78]. Therefore, for strongly suspected EoE cases or cases with persistent symptoms on-PPI therapy, re-evaluation of endoscopy and biopsy examination off-PPI therapy is necessary in some cases without esophageal eosinophilia. In cases with reflux symptoms without strong suspicions of EoE, diagnosis of GERD or PPI-refractory GERD can be made.

#### Functional GI disorders (FGIDs)

Rome IV criteria show the diagnostic criteria of esophageal and gastroduodenal disorders [69, 79]. Among these, all functional esophageal disorders, including functional chest pain, functional heartburn, reflux hypersensitivity, globus, and functional dysphagia, must be fulfilled with the absence of evidence that EoE is the cause of the symptoms [69]. In functional dyspepsia, there is no statement regarding EoE [79]. However, because patients with EoE frequently complain of epigastralgia or abdominal pain, EoE must represent a differential diagnosis, particularly for cases refractory to the standard therapy. Although other diagnostic modalities, such as esophageal high-resolution manometry (HRM) and impedance–pH monitoring, are required according to Rome IV criteria [69], cases with chest pain, heartburn globus sensation, dysphagia, or epigastralgia could be diagnosed as FGIDs according to symptoms when esophageal eosinophilia is absent.

#### EoE-like disease

Straumann et al. reported 5 cases with EoE-typical and corticosteroid-responsive symptoms without esophageal eosinophilia in 4 EoE families [80]. They found T-cell infiltration and no increase in eotaxin-3 levels—a key cytokine of EoE—in the esophagus and accordingly proposed a new disease entity [80]. A recent multicenter study identified 71 patients, of which half were female, 95% reported dysphagia, and 52% were endoscopically active. The study researchers found that the expression of lymphoepithelial Kazal-type-related inhibitor (LEKT1), a protease inhibitor responsible for epithelial homeostasis, was low compared to the control, albeit to a lesser extent than EoE [81]. Although EoE-like disease is one of the causes of cases with the absence of esophageal eosinophilia, a case of EoE-like disease has not yet been reported in Japan.

#### Eosinophilic esophageal myositis

Eosinophilic esophageal myositis (EoEM) was first reported by Sato [82] and was associated with Jackhammer esophagus as per the Chicago classification [83]. EoEM is defined as an eosinophilic infiltration in the esophageal muscle layer but not in the mucosa as well as the presence of EoE-like symptoms, such as dysphagia, food impaction, and chest pain [84, 85]. EoEM is a rare disease but must be differentiated in cases with EoE symptoms in the absence of esophageal eosinophilia. EoEM is suspected by HRM findings (Jackhammer esophagus) and confirmed by histological examination of the esophageal muscular layer by per-oral endoscopic myotomy with biopsy (POEM-b) [86] or endoscopic ultrasound-guided fine-needle aspiration biopsy [87]. Recently, Spechler proposed an interesting hypothesis that the clinical manifestations are determined by the layers exhibiting eosinophilic infiltration. Furthermore, they showed the possibility that EoE has mucosal-predominant and muscle-predominant forms and that the muscle-predominant form of EoE can cause various esophageal motility disorders including achalasia [88]. They also hypothesized that eosinophil infiltration in the esophageal mucosa could be found due to chronic contact of residue in patients with achalasia [89]. Further studies regarding the association between EoE or EoEM and esophageal motility disorders are warranted.

## Current treatment of EoE

**Fig. 6:**
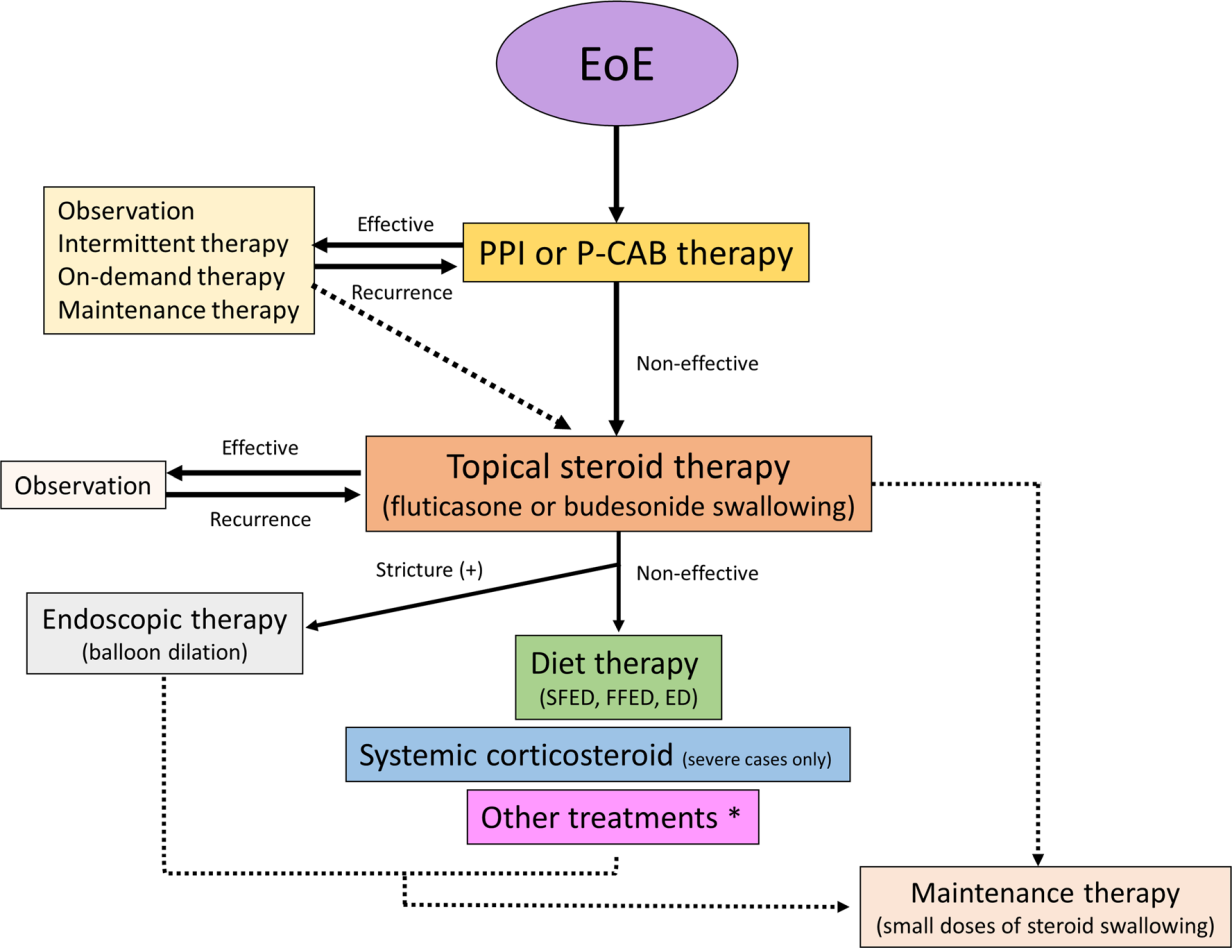
Current treatment strategy (*modified from reference no. 90*). *Other treatments include montelukast, sodium cromoglicate, immunosuppressive drugs, and biologics. *PPI* proton pump inhibitor, *P-CAB* potassium competitive acid blocker, *SFED* six-food-elimination diet, *FFED* four-food-elimination diet, *ED* elemental diet

The current treatment of EoE has briefly been outlined [1–3, 6–8, 90, 91], and it should be noted that the medications currently available for EoE have not been approved by medical insurance of the government in Japan. Because most cases of EoE in Japan are mild, PPI or P-CAB therapy is the first-line treatment; however, the doses and duration of these drugs remain to be clarified. In cases with improvement following PPI or P-CAB therapy, observation after therapy, intermittent therapy, on-demand therapy, or maintenance therapy are selected based on the severity of symptoms. Interestingly, this therapeutic strategy is similar to that of GERD treatment [92]. In cases where PPI or P-CAB therapy does not improve symptoms, topical steroid therapy is required. Fluticasone or budesonide is commonly used for steroid swallowing, and a low dose of steroid swallowing can be used as maintenance therapy if necessary. Systemic corticosteroid therapy is limited to severe cases that require hospitalization. Furthermore, the six or four food elimination diet is an important fundamental treatment because specific allergens can be identified, although it is difficult to perform for adults. Moreover, in cases with strictures, endoscopic balloon dilation is safe and effective. The current treatment strategy is summarized in Fig. 6 [91].

## Conclusion and diagnostic approach

**Fig. 7:**
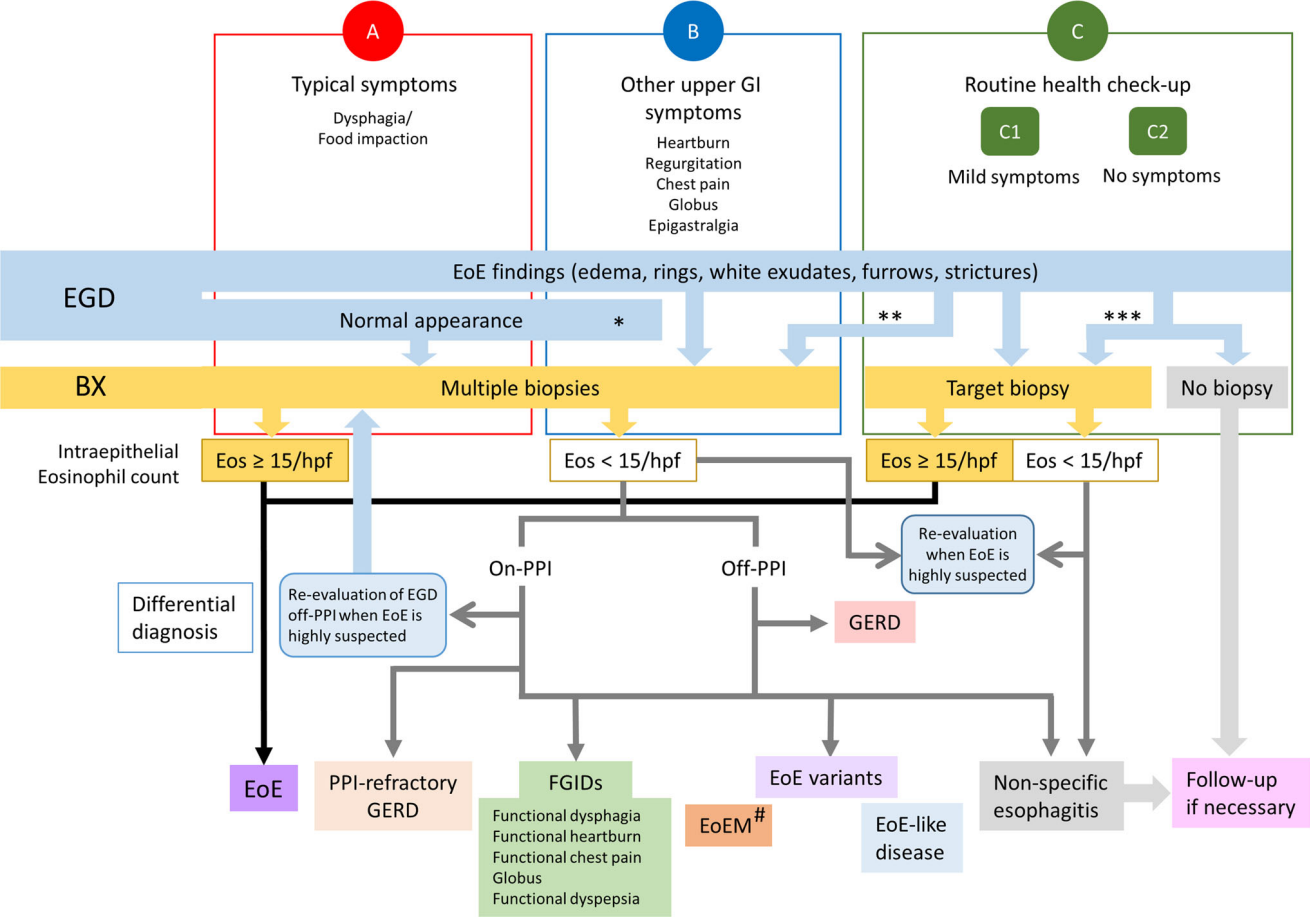
Symptom-based diagnostic approach for eosinophilic esophagitis. *Case refractory to standard treatment; **Refer to a high-volume center without biopsy; ***severe endoscopic findings or other reasons. *EGD* esophagogastroduodenoscopy, *BX* biopsy, *Eos* eosinophils, *GERD* gastroesophageal reflux disease, *EoE* eosinophilic esophagitis, *FGID* functional gastrointestinal disorder, *EoEM* eosinophilic esophageal myositis

This review has clarified the symptom variations and prevalence of EoE in Japanese adult patients and described several important considerations in the diagnosis of EoE. The review is summarized as a flowchart of the symptom-based diagnostic approach for EoE in Fig. 7.

## References

[1] Dellon ES, Gonsalves N, Hirano I (2013). ACG clinical guideline: evidenced based approach to the diagnosis and management of esophageal eosinophilia and eosinophilic esophagitis (EoE). Am J Gastroenterol.

[2] Lucendo AJ, Molina-Infante J, Arias Á (2017). Guidelines on eosinophilic esophagitis: evidence-based statements and recommendations for diagnosis and management in children and adults. United European Gastroenterol J.

[3] Dellon ES, Liacouras CA, Molina-Infante J (2018). Updated international consensus diagnostic criteria for eosinophilic esophagitis proceedings of the agree conference. Gastroenterology.

[4] Dellon ES, Hirano I (2018). Epidemiology and natural history of eosinophilic esophagitis. Gastroenterology.

[5] Kinoshita Y, Ishimura N, Oshima N (2015). Systematic review: eosinophilic esophagitis in Asian countries. World J Gastroenterol.

[6] Ishimura N, Kinoshita Y (2018). Eosinophilic esophagitis in Japan: Focus on response to acid suppressive therapy. J Gastroenterol Hepatol.

[7] Kinoshita Y, Ishihara S (2020). Eosinophilic gastroenteritis: epidemiology, diagnosis, and treatment. Curr Opin Allergy Clin Immunol.

[8] Haruma K (2019). Eosinophilic esophagitis: time for clinical practice. Esophagus.

[9] Dellon ES, Gibbs WB, Fritchie KJ (2009). Clinical, endoscopic, and histologic findings distinguish eosinophilic esophagitis from gastroesophageal reflux disease. Clin Gastroenterol Hepatol.

[10] Remedios M, Campbell C, Jones DM (2006). Eosinophilic esophagitis in adults: clinical, endoscopic, histologic findings, and response to treatment with fluticasone propionate. Gastrointest Endosc.

[11] Adachi K, Okada M, Mishiro T (2018). Clinical features and prognosis of eosinophilic esophagitis case diagnosed by upper GI endoscopic screening (in Japanese). Stomach and Intestine.

[12] Sato H, Honma T, Nozawa Y (2018). Eosinophilic esophagitis in Japanese patients: a mild and slow-progressing disorder. PLoS ONE.

[13] Tanaka F, Fukumoto S, Morisaki T (2019). Obesity and hiatal hernia may be non-allergic risk factors for esophageal eosinophilia in Japanese adults. Esophagus.

[14] Ishibashi F, Fukushima K, Onizuka R (2019). Risk of progression to eosinophilic esophagitis in patients with asymptomatic esophageal eosinophilia: a retrospective pilot study. J Gastroenterol Hepatol Open.

[15] Abe Y, Iijima K, Ohara S (2011). A Japanese case series of 12 patients with esophageal eosinophilia. J Gastroenterol.

[16] Fujishiro H, Amano Y, Kushiyama Y (2011). Eosinophilic esophagitis investigated by upper gastrointestinal endoscopy in Japanese patients. J Gastroenterol.

[17] Fujiwara Y, Sugawa T, Tanaka F (2012). A multicenter study on the prevalence of eosinophilic esophagitis and PPI-responsive esophageal eosinophilic infiltration. Intern Med.

[18] Kinoshita Y, Furuta K, Ishimaura N (2013). Clinical characteristics of Japanese patients with eosinophilic esophagitis and eosinophilic gastroenteritis. J Gastroenterol.

[19] Tomomatsu Y, Yoshino J, Inui K (2013). Clinical features of eosinophilic esophagitis ten Japanese cases. Dig Endosc.

[20] Hori K, Watari J, Fukui H (2014). Do endoscopic features suggesting eosinophilic esophagitis represent histological eosinophilia?. Dig Endosc.

[21] Abe Y, Iijima K, Ohara S (2014). Localized esophageal eosinophilia: Is it an early manifestation of eosinophilic esophagitis or a subtype of gastroesophageal reflux disease?. Dig Endosc.

[22] Shimura S, Ishimura N, Tanimura T (2014). Reliability of symptoms and endoscopic findings for diagnosis of esophageal eosinophilia in a Japanese population. Digestion.

[23] Kusunose H, Ohara S, Hamada S (2014). The investigation of clinical features of eosinophilic esophagitis and the possibility of acid related disease judged the effects of PPIs (in Japanese). Therapeutic Res.

[24] Iwakura N, Fujiwara Y, Tanaka F (2015). Basophil infiltration in eosinophilic oesophagitis and proton pump inhibitor-responsive oesophageal eosinophilia. Aliment Pharmacol Ther.

[25] Ishimura N, Ishihara S, Kinoshita Y (2016). Sustained Acid suppression by potassium-competitive acid blocker (P-CAB) may be an attractive treatment candidate for patients with eosinophilic esophagitis. Am J Gastroenterol.

[26] Jiao D, Ishimura N, Maruyama R (2017). Similarities and differences among eosinophilic esophagitis, proton-pump inhibitor-responsive esophageal eosinophilia, and reflux esophagitis: comparisons of clinical, endoscopic, and histopathological findings in Japanese patients. J Gastroenterol.

[27] Okimoto E, Ishimura N, Okada M (2017). Specific locations of linear furrows in patients with esophageal eosinophilia. Dig Endosc.

[28] Okimoto K, Arai M, Ishigami H (2018). A prospective study of eosinophilic esophagitis and the expression of tight junction proteins in patients with gastroesophageal reflux disease symptoms. Gut Liver.

[29] Ishimura N, Sumi S, Okada M (2018). Ankylosaurus back sign: novel endoscopic finding in esophageal eosinophilia patients indicating proton pump inhibitor response. Endosc Int Open.

[30] Ishimura N, Sumi S, Okada M (2019). Is Asymptomatic esophageal eosinophilia the same disease entity as eosinophilic esophagitis?. Clin Gastroenterol Hepatol.

[31] Sawada A, Hashimoto A, Uemura R (2019). Association between endoscopic findings of eosinophilic esophagitis and responsiveness to proton pump inhibitors. Endosc Int Open.

[32] Takashima S, Tanaka F, Otani K (2019). Barrett’s esophagus is negatively associated with eosinophilic esophagitis in Japanese subjects. Esophagus.

[33] Horiki N, Maruyama M, Fujita Y (1998). A case of idiopathic eosinophilic esophagitis with CT finding showing marked thickening of the esophageal wall (in Japanese). Nihon Shokakibyo Gakkai Zasshi.

[34] Furuta K, Adachi K, Kowari K (2006). A Japanese case of eosinophilic esophagitis. J Gastroenterol.

[35] Kamimura K, Oosaki A, Sugahara S (2008). Eosinophilic esophagitis: a case report Effective treatment with systemic corticosteroids for the relapse of the disease. Clin J Gastroenterol.

[36] Minematsu H, Hayafuji K, Saito Y (2009). A case of eosinophilic esophagitis with pleural effusion (in Japanese). Gastroenterol Endosc.

[37] Sano H, Iwakiri K, Kawami N (2010). Eosinophilic esophagitis: a case report with a review of the literature. Clin J Gastroenterol.

[38] Fukuchi M, Sakurai S, Suzuki M (2011). Esophageal squamous cell carcinoma with marked eosinophil infiltration. Case Rep Gastroenterol.

[39] Tamagawa Y, Miyake T, Mishiro T (2011). A case of eosinophilic esophagitis with atypical clinical course. Clin J Gastroenterol.

[40] Fujiwara Y, Muraki M, Kohata Y (2011). A case of eosinophilic esophagitis with a stricture successfully treated using fluticasone swallowing therapy. Gastroenterol Endosc.

[41] Maruyama Y, Kageoka M, Ohata A (2011). A case of eosinophilic esophagitis (in Japanese). Stomach Intestine.

[42] Tomomatsu Y, Yoshino J, Inui K (2011). A case of eosinophilic esophagitis (in Japanese). Stomach Intestine.

[43] Fukuchi M, Sakurai S, Fukasawa T (2012). Two Japanese patients with esophageal eosinophilia detected by routine medical examination. Esophagus.

[44] Takashima S, Inaba T, Ishikawa S (2013). Two cases of eosinophilic esophagitis with white exudates during endoscopy (in Japanese). Clini Dig Tract.

[45] Oda J, Iriguchi Y, Mizutani M (2013). A case of eosinophilic esophagitis (in Japanese). Stomach Small Intestine.

[46] Nakamura F, Yano M, Tamura U (2013). A case of eosinophilic esophagitis (in Japanese). Tokushima J Med.

[47] Tasaki S, Kawasaki T, Hayashi K (2013). A case of eosinophilic esophagitis with GERD-like symptoms (in Japanese). Prog Dig Endo.

[48] Yamabe A, Irisawa A, Shibukawa G (2014). Clinical effects of eosinophilic esophagitis observed using endoscopic ultrasound. Clin J Gastroenterol.

[49] Kondo T, Uehara T, Takada T (2015). Recurrent back pain of eosinophilic esophagitis. Am J Med.

[50] Kita Y, Tatewaki M, Furukawa M (2014). Six cases of eosinophilic esophagitis (in Japanese). Nigen Dock.

[51] Fujiwara Y, Iwakura N, Hashimoto A (2015). Successful treatment of localized eosinophilic esophagitis with a proton pump inhibitor (in Japanese). Gastroenterol Endosc.

[52] Sasaki Y, Harada M, Kiya Y (2015). A case of eosinophilic esophagitis, the diagnosis was difficult by endoscopic findings (in Japanese). Progress Dig Endo.

[53] Fukuoka K, Izumi T, Yaku H (2015). A case of eosinophilic esophagitis caused by a cedar ball (in Japanese). Nihon Shokakibyo Gakkai Zasshi.

[54] Miyaoka Y, Tsukano K, Ueno S (2015). A case of eosinophilic esophagitis complicated with Crohn’s disease (in Japanese). Gastroenterol Endosc.

[55] KitamuraH HT (2016). Two cases of eosinophilic esophagitis (in Japanese). Jap J Clin Radiol.

[56] Kawashima K, Ishihara S, Masuhara M (2018). Development of eosinophilic esophagitis following sublingual immunotherapy with cedar pollen extract: a case report. Allergol Int.

[57] Matsuura H, Muro S, Yamauchi K (2018). Eosinophilic esophagitis: crepe paper-like appearance. Am J Med.

[58] Tanaka S, Toyonaga T, Kawara F (2018). A case of Jackhammer esophagus caused by eosinophilic esophagitis in which per-oral endoscopic myotomy resulted in symptom improvement. Clin J Gastroenterol.

[59] Mitani K, Kubo T, Saito T (2018). A case of eosinophilic esophagitis developed after cessation of ICS/LTRA therapy (in Japanese). Med J Nara Pref Western Med Center.

[60] Kinugasa H, Tanaka T, Okada H (2019). Esophageal melanosis with eosinophilic esophagitis. Gastrointest Endosc.

[61] Owaki T, Sato H, Horigome R (2019). Eosinophilic esophagitis after total gastrectomy treated with proton pump inhibitors: a case report. Clin J Gastroenterol.

[62] Yamazaki K, Kojima K, Iwata H (2019). Eosinophilic Esophagitis Mimicking Candida Esophagitis. Intern Med.

[63] Kojima S, Katsuhara M, Takeuchi T (2019). A case of eosinophilic esophagitis that transformed from the localized type to the diffuse type (in Japanese). Gastroenterol Endosc.

[64] Fujiwara Y, Hashimoto A, Uemura R (2019). Optimal biopsy protocol to evaluate histological effectiveness of proton pump inhibitor therapy in patients with eosinophilic esophagitis. Digestion.

[65] Hirano I, Moy N, Heckman MG (2013). Endoscopic assessment of the oesophageal features of eosinophilic oesophagitis: validation of a novel classification and grading system. Gut.

[66] Hirano I (2020). Clinical relevance of esophageal subepithelial activity in eosinophilic esophagitis. J Gastroenterol.

[67] Izumi D, Ishimura N, Okada M (2017). Poor inter-observer agreement on the endoscopic diagnosis of eosinophilic esophagitis among Japanese endoscopists. Esophagus.

[68] Fujiwara Y, Tanigawa T, Yamagami H (2013). Eosinophilic esophagitis-like endoscopic findings in patients with erosive esophagitis. Esophagus.

[69] Aziz Q, Fass R, Gyawali CP (2016). Functional Esophageal Disorders. Gastroenterology.

[70] Fass R (2009). Proton pump inhibitor failure–what are the therapeutic options?. Am J Gastroenterol.

[71] Kawami N, Takenouchi N, Umezawa M (2017). Pathogenesis of double-dose proton pump inhibitor-resistant non-erosive reflux disease, and mechanism of reflux symptoms and gastric acid secretion-suppressive effect in the presence or absence of helicobacter pylori infection. Digestion.

[72] Adachi K, Mishiro T, Tanaka S (2016). Suitable biopsy site for detection of esophageal eosinophilia in eosinophilic esophagitis suspected cases. Dig Endosc.

[73] Fujiwara Y, Arakawa T (2009). Epidemiology and clinical characteristics of GERD in the Japanese population. J Gastroenterol.

[74] Kohata Y, Fujiwara Y, Kato K (2014). Successful treatment of betamethasone syrup on autoimmune esophagitis. Am J Gastroenterol.

[75] Nakamura R, Omori T, Suda K (2017). Endoscopic findings of laryngopharyngeal and esophageal involvement in autoimmune bullous disease. Dig Endosc.

[76] Toya Y, Nakamura S, Tomita K (2016). Dabigatran-induced esophagitis: the prevalence and endoscopic characteristics. J Gastroenterol Hepatol.

[77] Matsushita T, Maruyama R, Ishikawa N (2015). The number and distribution of eosinophils in the adult human gastrointestinal tract: a study and comparison of racial and environmental factors. Am J Surg Pathol.

[78] Odiase E, Schwartz A, Souza RF (2018). New eosinophilic esophagitis concepts call for change in proton pump inhibitor management before diagnostic endoscopy. Gastroenterology.

[79] Stanghellini V, Chan FK, Hasler WL (2016). Gastroduodenal disorders. Gastroenterology.

[80] Straumann A, Blanchard C, Radonjic-Hoesli S (2016). A new eosinophilic esophagitis (EoE)-like disease without tissue eosinophilia found in EoE families. Allergy.

[81] Greuter T, Simon D, Collins MH (2019). Eosinophilic esophagitis-like disease with lack of significant esophageal eosinophilia: Description of a new disease entity. Gastroenterology.

[82] Sato H, Takeuchi M, Takahashi K (2015). Eosinophilic infiltration of the muscularis propria in a patient with jackhammer esophagus treated with per-oral endoscopic myotomy. Clin Gastroenterol Hepatol.

[83] Kahrilas PJ, Bredenoord AJ, Fox M (2015). The Chicago classification of esophageal motility disorders, v.30. Neurogastroenterol Motil.

[84] Sato H, Nakajima N, Hasegawa G (2017). Immunohistochemical differentiation of eosinophilic esophageal myositis from eosinophilic esophagitis. J Gastroenterol Hepatol.

[85] Sato H, Nakajima N, Takahashi K (2017). Proposed criteria to differentiate heterogeneous eosinophilic gastrointestinal disorders of the esophagus, including eosinophilic esophageal myositis. World J Gastroenterol.

[86] Sato H, Terai S (2018). Eosinophilic esophageal myositis (EoEM) causes jackhammer esophagus, rarely posing a problem in the differential diagnosis of eosinophilic esophagitis. Am J Gastroenterol.

[87] Igarashi R, Irisawa A, Shibukawa G (2016). Eosinophilic esophageal myositis diagnosed by endoscopic ultrasound-guided fine-needle aspiration biopsy: a case report. Clin J Gastroenterol.

[88] Spechler SJ (2019). Eosinophilic esophagitis: novel concepts regarding pathogenesis and clinical manifestations. J Gastroenterol.

[89] Spechler SJ, Konda V, Souza R (2018). Can eosinophilic esophagitis cause achalasia and other esophageal motility disorders?. Am J Gastroenterol.

[90] Philpott H, Dellon ES (2018). The role of maintenance therapy in eosinophilic esophagitis: who, why, and how?. J Gastroenterol.

[91] Fujiwara Y, Hashimoto A (2018). Treatment for eosinophilic esophagitis (in Japanese). Stomach Intestine.

[92] Iwakiri K, Kinoshita Y, Habu Y (2016). Evidence-based clinical practice guidelines for gastroesophageal reflux disease 2015. J Gastroenterol.

